# Europe gears up to attend to refugees’ health

**DOI:** 10.2471/BLT.15.021215

**Published:** 2015-12-01

## Abstract

Countries already squeezed by the financial crisis are struggling to respond to the health needs of large numbers of refugees and migrants. Andréia Azevedo Soares and Menelaos Tzafalias report.

Dr Santino Severoni and his team conducted their first mission to Lampedusa in March 2011. For months large numbers of refugees and migrants from the Eastern Mediterranean, Africa and Asia had been arriving on the shores of the tiny Italian island.

“We realized back then that few if any countries had considered the scenario of a large influx of refugees and migrants to Europe,” says Severoni, public health and migration coordinator at the World Health Organization (WHO) Regional Office for Europe.

“Countries were prepared for disasters such as earthquakes or floods, but the sudden arrival of a massive group of refugees was hardly considered,” he says, “let alone the health consequences of such an influx”.

Four years later that scenario has become a stark reality for many European countries.

The large numbers of refugees and migrants arriving since then have put a major strain on European countries that are still feeling the effects of the 2008 financial crisis. 

An estimated 700 000 refugees and migrants arrived in the Europe Union this year and, according to the European Commission, more than three million more may arrive by the end of 2016.

Severoni and his team have been working on a project to help such countries. 

A key element of this project – known as PHAME or “Public Health Aspects of Migration in Europe” – is the technical support provided by WHO to these countries. 

This involves a mission to each country concerned to assess the health system’s ability to cope with the arrival of large numbers of people and make recommendations in a report. Severoni’s team have designed a “toolkit” or guide for this purpose. 

Greece has become the main entry point for those travelling to Europe in recent months. More than half a million refugees and migrants have arrived in Greece since the beginning of this year, according to the United Nations High Commissioner for Refugees (UNHCR).

The vast majority of them are refugees, often traumatized families fleeing the conflict in the Syrian Arab Republic followed by other refugees from Afghanistan, Iraq and Pakistan. The rest are migrants, people seeking better living conditions in another country. 

Medécins Sans Frontières (MSF) is one of several nongovernmental organizations providing medical and mental health care services to the new arrivals on many Greek islands and on the border with the former Yugoslav Republic of Macedonia. 

“Everywhere we work, the local health centres and hospitals are struggling because they are already serving the local population and the holidaymakers – and now refugees,” says Constance Theisen, humanitarian affairs officer with MSF in Greece, where MSF physicians make referrals to local health services.

“We do medical consultations everywhere we work. We also offer mental health support and have a psychologist in all our teams,” says Theisen. 

In the vast majority of cases – whether people are receiving first aid or mental health support – it is short-term because most of them want to continue to their destination countries. 

“Even when we have serious incidents, when a medical evacuation to a hospital is necessary, the migrants themselves do not want to go to hospital, they just want to move on,” says Dr Hasan Abuhamdan, working with MSF in Greece at the Eidomeni temporary relief camp on the border with the former Yugoslav Republic of Macedonia. 

The WHO toolkit has been piloted successfully in many countries on Europe’s southern rim, including in Albania, Bulgaria, Cyprus, Greece, Hungary, Italy, Malta, Portugal, Serbia and Spain.

“Our team arrives in the country and we assess the national health system by interviewing all key stakeholders involved in the management of the migration process in the country, in collaboration with the ministry of health visiting the most important spots and institutions receiving the migrants,” Severoni says.

“We usually find that countries do have the capacity to deal with the situation, but they are not always prepared,” says Severoni, adding: 

“That’s why we encourage them to prepare contingency plans to respond to the health aspects of these influxes and establish a clear hierarchy for the partners involved. That way the response can be as effective and efficient as possible in the given time frame.”

In December 2014, before very large numbers of people started arriving in Greece, Severoni and his team conducted an assessment with the Greek government to gauge the country’s level of preparedness and the capacity of local health systems to respond.

“Greece faces challenges in terms of coordinating the institutions and organizations responsible for the refugees and responding to their needs,” says Eleni Antoniadou, advisor to the president of the Hellenic Centre for Disease Control and Prevention.

Greece’s health ministry and public health protection agency – the Hellenic Centre for Disease Control and Prevention – have been taking the lead in collaboration with intergovernmental agencies, nongovernmental organizations (NGOs) and volunteer groups, as well as international organizations such as WHO and UNHCR.

“We need substantial financial assistance to be able to respond better to the increasing numbers of refugees arriving,” says Antoniadou, adding that Greece has applied to the European Commission for emergency funding for a health response plan drawn up by the Hellenic Centre for Disease Control and the health ministry.

From the islands, the Greek authorities transfer the new arrivals to the mainland, where most continue their journey through the former Yugoslav Republic of Macedonia, Serbia, Hungary and Austria. The aim is to reach Germany, Sweden and other countries in the north.

The poor state of the refugees’ and migrants’ health is often a result of their long and exhausting journey. 

“Most Greek islands are not offering them any type of shelter when they arrive. They usually arrive completely drenched because the last part of the journey is by sea.

“They don't eat properly, they have limited access to drinking water and hygiene facilities – often no shower or toilets – and this all affects their health,” Theisen says.

In October Severoni’s team trained more than 150 medical professionals and Red Cross volunteers in the former Yugoslav Republic of Macedonia to respond to the health needs of the refugees and migrants.

In addition, WHO’s Regional Office for Europe has provided medicines and other medical supplies to several other transit countries, such as Greece and Serbia.

In the WHO–health ministry assessment in Portugal, on Europe’s Atlantic rim, the country was praised for its laws granting undocumented migrants access to health care.

“We need to act beyond the emergency and focus on preparedness and resilience.”

Santino Severoni

Some 191 refugees and 1309 migrants are expected to be resettled from Greece and Italy to Portugal, as agreed during European Union talks this year.

But even in Portugal "some grey areas persist in [the application of] immigration law", according to the assessment of the WHO mission and the health ministry.

“The joint mission helped us to identify inefficiencies in the health system,” says Eva Falcão, director of the Directorate of International Relations at the Portuguese Directorate-General of Health.

For example, the mission found that conflicting interpretations of the legislation among administrative staff were limiting access to health services for undocumented migrants. "This can be only solved with training and dialogue,” says Falcão.

To some extent, each country is taking its own approach.

“A few years ago Spain was considered a champion of health equity and integration,” says Davide Mosca, director of the Migration and Health Division at the International Organization for Migration, referring to the time before 2012 when legislation was introduced that excluded nearly a million undocumented people from the national health system.

“No doubt this change is the effect of the financial crisis,” Mosca adds.

Over the border with Greece, in the town of Gevgelija, in the former Yugoslav Republic of Macedonia, the Red Cross and the UNHCR are providing food, water, clothes and medical care and support to refugees and migrants arriving at the Vinojug transit centre.

“The number of times medical assistance [was provided] exceeded 1000 per day in September and October of this year,” says Snezhana Chichevalieva, head of the WHO Country Office in the capital Skopje. 

 “Mostly they treat skin problems such as burns and blisters, allergic reactions and insect bites, pain as well as wounds related to the conflicts they fled, respiratory and gastro-intestinal diseases, and chronic diseases such as diabetes and heart disease,” Chichevalieva says.

The emergency unit at the health-care centre in Kumanovo provides emergency care and transport of patients to the city’s general hospital, she says. 

“Most patients are not willing to go to the hospital when they are referred because they want to leave the country as soon as possible.”

For Mosca, Europe’s migrant and refugee crisis may set the tone for decades to come. “With migration and human mobility as mega-trends of the 21st century, the health of migrants needs to be seen as a global health issue. Health systems need to be prepared to this new reality.”

Severoni and his team are hoping that useful lessons can be drawn from the current situation so that countries can be better prepared for the arrival of large numbers of people in future.

 “We need to act beyond the emergency and focus on preparedness and resilience”.

**Figure:**
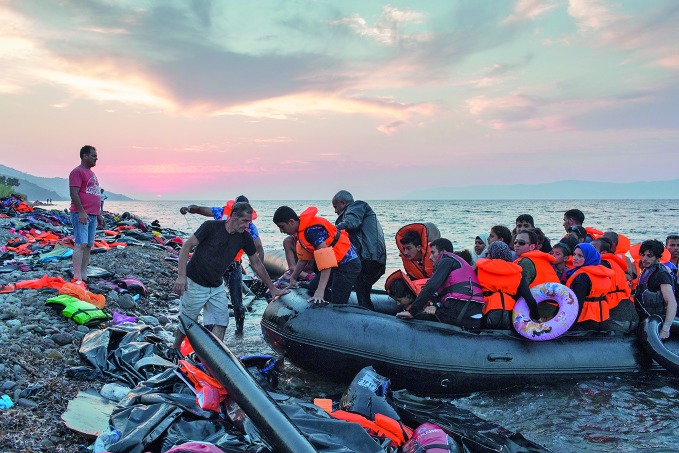
Refugees arrive on the Island of Lesbos, Greece, after crossing the Aegean Sea from Turkey.

UNCHR/I. Prickett

**Figure:**
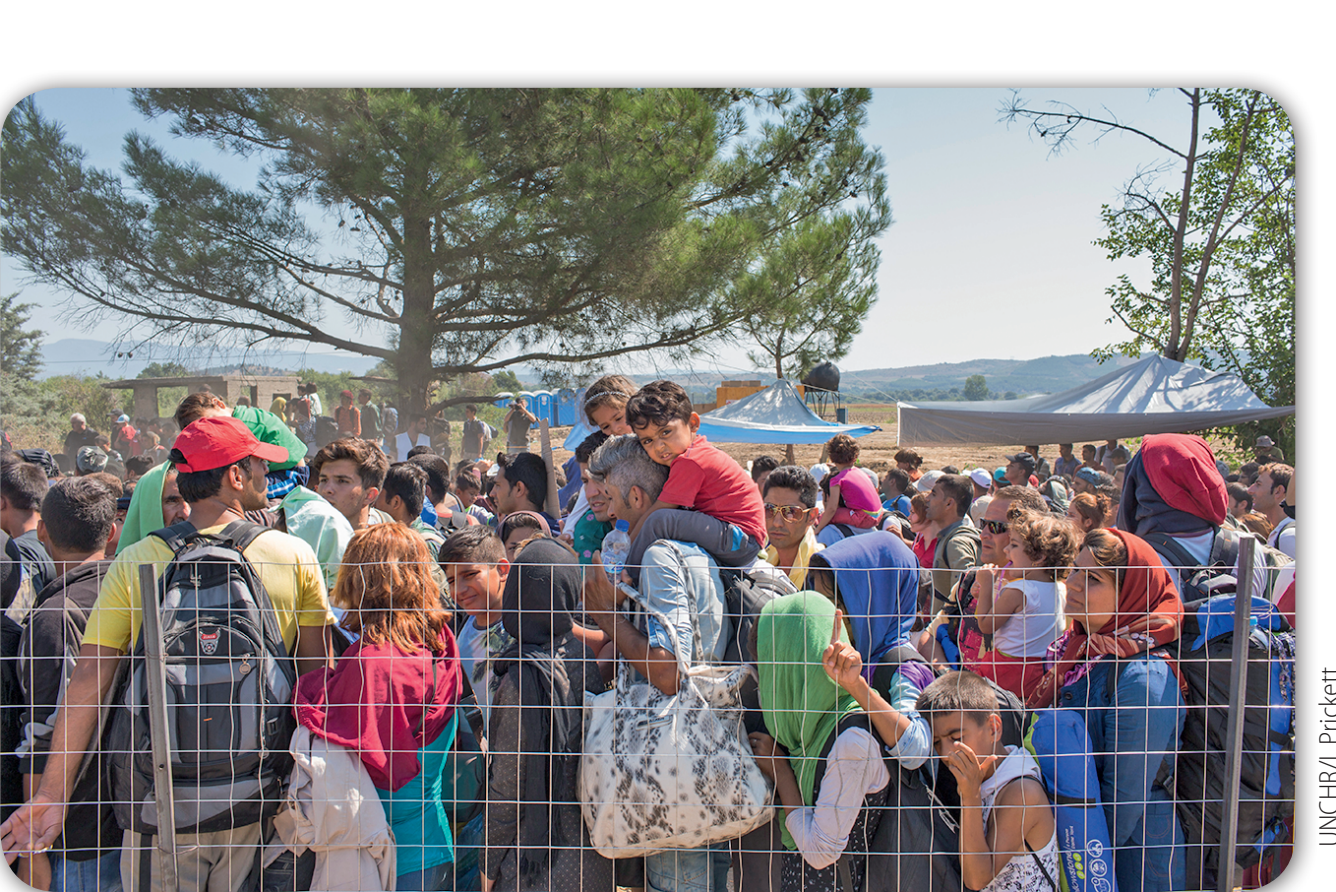
Refugees and migrants wait at the Greek border to cross into the former Yugoslav Republic of Macedonia.

UNCHR/I. Prickett

